# What makes the hepatitis C virus evolve?

**DOI:** 10.7554/eLife.50148

**Published:** 2019-09-03

**Authors:** Thomas R O'Brien, Rune Hartmann, Ludmila Prokunina-Olsson

**Affiliations:** 1 Infections and Immunoepidemiology Branch, Division of Cancer Epidemiology and GeneticsNational Cancer Institute, National Institutes of HealthBethesdaUnited States; 2 Department of Molecular BiologyUniversity of AarhusAarhusDenmark; 3 Laboratory of Translational Genomics, Division of Cancer Epidemiology and GeneticsNational Cancer Institute, National Institutes of HealthBethesdaUnited States

**Keywords:** Hepatitis C, host-pathogen interactions, interferon-lambda 4, innate immunity, Human, Virus

## Abstract

Polymorphisms in the *IFNL4* gene that affect both the presence and the form of the coded protein are associated with changes in the hepatitis C virus.

**Related research article** Chaturvedi N, Svarovskaia ES, Mo H, Osinusi AO. 2019. Adaptation of hepatitis C virus to interferon lambda polymorphism across multiple viral genotypes. *eLife* **8**:e42542. doi: 10.7554/eLife.42542

**Related research article** Ansari MA, Aranday-Cortes E, Ip CLC, da Silva Filipe A, Hin LS, Bamford CGG, Bonsall D, Trebes A, Piazza P, Sreenu V, Cowton VM, STOP-HCV Consortium, Hudson E, Bowden R, Patel AH, Foster GR, Irving WL, Agarwal K, Thomson EC, Simmonds P, Klenerman P, Holmes C, Barnes E, Spencer CCA, McLauchlan J, Pedergnana V. 2019. Interferon lambda 4 impacts the genetic diversity of hepatitis C virus. *eLife* **8**:e42463. doi: 10.7554/eLife.42463

When cells are infected with a virus they release proteins known as interferons to fight off the infection. In 2003, a group of proteins called interferon lambda, or IFN-λ for short, were found to activate anti-viral signaling pathways used by other interferons (Kotenko et al., 2003; Sheppard et al., 2003). It was initially thought that members of the IFN-λ family were simply redundant to well-known interferons. However, a growing body of evidence indicates IFN-λ proteins provide unique ‘first-line’ protection against a wide range of common viruses that can infect the respiratory and gastrointestinal tracts, as well as the brain (Ye et al., 2019).

IFN-λ4 is the most recently discovered member of the IFN-λ family. Production of IFN-λ4 in humans depends on which variant of the *IFNL4* gene a person carries. Individuals with the *IFNL4*-ΔG variant can synthesize the full protein, while those with the *IFNL4*-TT variant produce a truncated protein that is not functional (Prokunina-Olsson et al., 2013). Another polymorphism called *IFNL4* P70S produces variants of the full protein with different biological activity: if the amino acid in position 70 is a proline, the protein is fully active, but if it is a serine the protein has reduced biological activity (Terczyńska-Dyla et al., 2014). These *IFNL4* genetic variants affect clearance of the hepatitis C virus, and have been associated with the risk of other conditions. These conditions include a rare form of ovarian cancer, more aggressive prostate cancer and liver inflammation and scarring caused by the hepatitis B and C viruses or non-alcoholic fatty liver disease (Eslam et al., 2015; Kelemen et al., 2015; Minas et al., 2018).

Worldwide, an estimated 71 million people are infected with hepatitis C virus. There are various genotypes and subtypes of HCV and, like other RNA viruses, HCV frequently mutates and can undergo selection for different viral variants. This genetic variability is clinically significant, as some changes in the HCV genome may impair the effectiveness of certain treatments. Paradoxically, although interferons are usually antiviral proteins, patients with the most biologically active form of IFN-λ4 are least likely to clear HCV. On the other hand, patients with the *IFNL4*-TT allele that do not produce active IFN-λ4 have the highest rates of spontaneous clearance and response to treatment (Prokunina-Olsson et al., 2013; Terczyńska-Dyla et al., 2014).

Now, in eLife, two collaborations report on the effect of *IFNL4* genotype on the HCV viral genome. Previously, in 2017, Azim Ansari, Vincent Pedergnana and colleagues at the University of Oxford and other institutions reported the results of a genome-to-genome analysis which revealed that the HCV genome evolves in response to the genome of the infected individual (Ansari et al., 2017). The analysis showed that HCV viral levels (number of virus particles) and the amino acid sequence of viral proteins depended on whether the patient had the *IFNL4*-TT or the *IFNL4*-ΔG gene variant. In the new eLife papers, researchers based in Oxford, Lausanne and elsewhere examine the association between host *IFNL4* genotype and viral genome variations more broadly.

In one paper, Ansari, Pedergnana and co-workers – including Elihu Aranday-Cortes and John McLauchlan of the MRC-University of Glasgow Centre for Virus Research – report that the presence of IFN-λ4, and also its biological activity, both affect how HCV evolves (Ansari et al., 2019). Ansari et al. analyzed the *IFNL4* genotypes of 485 patients with chronic HCV and the genomes of the virus with which they were infected. To avoid confounding factors, the analysis was restricted to patients of white ancestry infected with HCV genotype 3a. In this new work they show that in addition to the effect of the *IFNL4-*ΔG/TT variant, the *IFNL4* P70S polymorphism, which modulates IFN-λ4 activity, affects the amino acid sequence of HCV.

In the other eLife paper, Nimisha Chaturvedi, Jacques Fellay (both of the EPFL and the Swiss Institute of Bioinformatics) and colleagues at Gilead Sciences and Goethe University Hospital report the results of a study that included 8729 patients who were infected with a range of HCV genotypes (Chaturvedi et al., 2019). This investigation revealed that the polymorphism rs12979860, which serves as a marker for *IFNL4-*ΔG/TT, is associated with amino acid changes in viral proteins across viral genotypes. Different viral polymorphisms were affected in the different viral genotypes. Taken together these new results show IFN-λ4 drives hepatitis C evolution across multiple viral genotypes and that not simply the presence, but also the form of IFN-λ4 contributes to this effect.

How IFN-λ4 makes HCV evolve remains unknown. Future studies should attempt to elucidate this mechanism, although currently such work is hampered by the transient and cell-specific expression of *IFNL4* in human tissues, and the lack of animal models – neither mice nor rats carry the gene. As the IFN-λ family is known to play a role in a wide range of viral infections, it would be interesting to see whether variants of the *IFNL4* gene affect response to those infections or can drive the evolution of viruses other than HCV. The hypothesis that *IFNL4* genotype affects infections other than HCV is further supported by evidence showing that there has been strong evolutionary selection for *IFNL4*-TT, the variant of the gene that produces an inactive truncated protein (Key et al., 2014).

## Disclaimer

The content of this publication does not necessarily reflect the views or policies of the Department of Health and Human Services, nor does mention of trade names, commercial products, or organizations imply endorsement by the US Government.
